# A lower gender equality consciousness in rural and left-behind children: a latent class analysis

**DOI:** 10.3389/fpsyg.2024.1368023

**Published:** 2024-07-05

**Authors:** Yifei Li, Jingping Zhang, Jie Zhang, Juan Li, Yiping Chen, Man Zuo

**Affiliations:** ^1^Xiangya Nursing School of Central South University, Changsha, Hunan, China; ^2^Hunan University of Chinese Medicine, Changsha, Hunan, China; ^3^Department of Gastroenterology, Shenzhen Hospital, Southern Medical University, Shenzhen, Guangdong, China; ^4^Heyuan People’s Hospital, Guangdong, China

**Keywords:** school-age children, children, gender equality consciousness, gender role, latent class analysis

## Abstract

**Objective:**

School age is a critical period for the development of individual gender equality consciousness. The purpose of this study was to explore the potential classes of school-age children’s gender equality consciousness, influencing factors and their differences in gender role, thus providing targeted guidance for the formulation and implementation of gender equality education strategies.

**Methods:**

A cross-sectional survey was conducted among 1846 school-age children using the demographic information questionnaire, gender equality consciousness questionnaire and Bem Sex Role Inventory. A latent class analysis was performed to explore gender equality consciousness latent classes. Multinomial logistic regression analysis was conducted to examine the predictors of class membership, and chi-square test was used to compare the gender role of each latent class.

**Results:**

The average age of the included 1846 participants was 10.10 ± 1.82 years old. The proportion of boy, grade 6 and living in urban area, respectively, were 50.8, 25.3, and 60.2%. The only children was 16.3% and left-behind children was 22.5%. 60.5% of all children thought their parents had a good relationship. The core family structure in all participants was 54.1%. Mothers were the caregivers of most children (63.6%). The same-sex friends more than 3 was 73.5%, while opposite-sex friends ranged from 0 to 1 was 41.7%. Three latent classes were identified and labeled “high gender equality consciousness” class (20.6%), “moderate gender equality consciousness” class (42.3%) and “low high gender equality consciousness” class (37.1%). Factors affecting the different types of school-age children’s gender equality consciousness include gender, grade, caregiver, place of residence, whether they are left-behind children and parental relationship. Rural and left-behind children are more likely to enter the “low gender equality consciousness” group. Children in the “low gender equality consciousness” group had a lower proportion of androgynous gender role.

**Conclusion:**

Rural children and left-behind children are the priority groups for gender equality education. Gender role is the important predictors and intervention targets of children’s gender equality consciousness. Educators or policy makers can formulate targeted intervention measures according to the influencing factors of potential classes.

## 1 Introduction

Gender equality is one of the important goals of social progress and sustainable human development. Studies have found that high levels of gender equality can improve life expectancy and mental health, reduce depression, and contribute to a harmonious social environment for both men and women (Kolip et al., 2019; Heinz et al., 2020; Milner et al., 2020). How to improve the gender equality index of countries and regions has long been one of the key concerns of the United Nations. Field theory holds that an individual’s actions are affected by the field in which the individual is located (Bourdieu, 2004). Gender equality environment is a unique social field, and the gender equality consciousness of individuals in this field tends to have the same characteristics. At the same time, individual gender equality consciousness can also play a role in the construction of gender equality field in their region to a certain extent (Liuji, 2009). Therefore, raising individual gender equality consciousness has become one of the important measures to promote national and regional gender equality.

Gender equality consciousness refers to people’s perception and attitude toward the equality of rights and obligations between men and women in various fields (Su et al., 2020). Previous studies have found that school-age children are in a critical period for the development and transformation of individual gender equality consciousness (Banse et al., 2010; Siyanova-Chanturia et al., 2015). School age refers to the period from primary school to adolescence when children begin to develop (Wang, 2013), when children’s gender equality consciousness is highly malleable. The implementation of good gender equality education for school-age children will directly affect the correct establishment of social gender values for these children in the future (Tang et al., 2011). However, compared with developed countries such as the Netherlands and Sweden, China’s gender equality education started late and has not yet formed a relatively complete gender equality education system (Qu, 2022). Moreover, research on gender equality consciousness among children and adolescents has neither proposed criteria for distinguishing between different levels of children nor provided relevant references. In this case, the description of average scores alone may be too simplistic to differentiate between subgroups of children with different levels of gender equality consciousness, which would be detrimental to the subsequent educational formation, and therefore a “person-centered” approach is more appropriate. However, the current research on the gender equality consciousness of children and adolescents in China mainly adopts the variable-centered analysis method, ignoring the heterogeneity of individuals.

LCA is a person-centered research methodology that determines the subgroups to which individuals belong by the response patterns of heterogeneous groups on epistatic variables (Ding, 2018). LCA captures group heterogeneity that is unobservable in variable-centered studies, and its objectivity in evaluating categorical indicators avoids, as far as possible, high within-category heterogeneity resulting from subjective categorical criteria (Zhang et al., 2017). Though cluster analysis and latent class analysis (LCA) are both used to classify individuals according to the distance or similarity between subjects, LCA can quantify of the uncertainty of class membership and assess the fit goodness (Dalmartello et al., 2020). Meanwhile, Tang et al. (2011) firstly assess gender equality consciousness through multiple dimensions, including family, occupation, and school, which probably contributes to quantify different levels of gender equality awareness. Therefore, LCA may objectively identify the latent classes of gender equality consciousness among different types of school-age children and analyze the relationship between classes and the potential factors suggested in previous researches (Valtolina and Colombo, 2012; Bleidorn et al., 2016; Matud et al., 2019; Luijten et al., 2023).

Variation in gender equality consciousness is affected by the changes of sex, grade, interpersonal relationship, and developing environment over school-age growing. Among school-age children, gender equality consciousness improved with each grade. Influenced by family support, boys and only children will show a higher gender equality consciousness; good peer relationships also improve subjective well-being (Li et al., 2021). Conversely, left-behind children are suffering various psychological trauma, such as depression, anxiety and loneliness (Wang et al., 2015). The group of “left-behind children” in rural hometowns emerged because a large amount of rural laborers flooded into urban areas (Duan and Zhou, 2005). Left-behind children, namely children who are under 18 years old and live at rural home but both or one of their parents leave rural areas for work for at least 6 months (Shen et al., 1998). Chinese left-behind children in rural areas showed a lower self-esteem score, which affected their ability of problem-solving and help-seeking (Cui et al., 2021). Although these predictors do not cover all variables that might involve in gender equality consciousness in school-age children, they offer a broad range of variables likely to help explain its potential reasons.

Other factor we explored was gender role in school-age children. Gender role is a behavioral norm corresponding to one’s own gender that individuals acquire through imitative learning in the process of socialization, and which vary according to different societies and cultures (Bem and Estes, 1981). It is an important part of an individual’s socialization as well as an integral part of the individual’s personality (Bem, 1974). Previous studies have found that individuals with the androgynous gender role tend to have stronger self-esteem, subjective well-being, social adaptability and gender equality consciousness due to the good qualities of both masculinity and femininity (Geng and Zhang, 2012; Yu et al., 2020; Li et al., 2021). As an important factor influencing the development of children’s psychological health, the gender role has a significant impact on school-age children’s gender equality consciousness, but the exact impact of different gender role on children’s gender equality consciousness is uncertain. Therefore, given the potentially critical role of gender role in raising gender equality consciousness among school-age children, it is necessary to compare the impact of gender role on different types of children’s gender equality consciousness.

Thus, this study employed LCA to (a) identify the latent classes in school-age children’s gender equality consciousness, (b) analyze the variables of latent classes memberships, and (c) compare the gender role of different latent classes, thus for school-age children’s gender equality education strategy formulation and implementation to provide targeted guidance.

## 2 Methods

### 2.1 Design

This study was a cross-sectional study conducted from March 2022 to June 2022 in six primary schools in the provinces of Henan, China.

### 2.2 Participants

To assure the characteristics diversity of the subjects, the participants were recruited from six screened primary schools in Henan Province, China. The screening criteria were as follows: (1) students had to be in grades first through sixth and were willing to voluntarily participate in the study; (2) had their parents’ consent; and (3) had not participated in other studies on gender equality and mental health of children.

### 2.3 Sample size

PASS15 software was used to estimate the sample size. Previous study (Li et al., 2021) showed that the total mean score of gender equality consciousness of school-age children was (17.29 ± 8.04), which meant that the overall standard deviation σ was 8.04. According to previous study (Ma et al., 2023), the confidence level is 0.95, the significance level α is 0.05, and the allowable error δ is 0.5, which are substituted into the formula n = (u_α_*σ/δ)^2^. At the same time, considering the invalid responses during the answering process, the response rate was set to 80%, and the sample size was calculated to be 1,193 people, which met the minimum sample size requirement of LCA (Meyer and Morin, 2016). A total of 1846 participants were included, which met the aforementioned sample size requirements.

### 2.4 Instruments

#### 2.4.1 Demographic information

Participants were asked to report their demographic information including gender (dichotomous question), age, grade, place of residence, whether you are the only child, whether your father or mother works outside the home, parents’ relationship, parental quarrels, family structure, caregiver, the number of same -sex friends, the number of opposite-sex friends, etc.

#### 2.4.2 Gender equality consciousness questionnaire

The gender equality consciousness questionnaire was developed by Tang et al. (2011). The questionnaire contains 30 items, involving the respondents’ attitudes toward gender equality in family, occupation and school. Each part contains 10 questions. Each question included three options: “Male,” “female,” and “both sexes.” The choice of “both sexes” is worth 1 point, and the remaining answers are not worth any point. Cronbach’s α was 0.92 and McDonald’s omega (HA) was 0.918 for this study.

#### 2.4.3 Gender role scale

Bem Sex Role Inventory (BSRI) was designed by Bem (1974) and translated by Lu and Su (2003), which consists of three scales: one measuring masculinity, one measuring femininity and finally one measuring social desirability. It is a 7-point scale (1–7, representing from “never true” to “always true”) to measures sex role orientation. The final measurements included masculinity, femininity, androgynous and undifferentiated. BSRI has an internal consistency and test–retest reliability around 0.80. Cronbach’s α was 0.91 and McDonald’s omega (HA) was 0.909 for this study.

#### 2.4.4 Missing data

Once a missing value occurs in any of the variables, the participant was excluded from the analysis. Age, gender, and other key variables between the studied and excluded population had no statistically difference.

### 2.5 Statistical analysis

Descriptive statistics were computed by SPSS 25.0. LCA and related analyses were computed by M-plus 8.0 software. All hypothesis tests were two-sided with a significance level of *α* = 0.05. Counting data is expressed as frequency (percentage). Multivariate Logistic regression analysis was used for multivariate analysis. Chi-square test was used for comparison between groups. Statistical indicators for LCA include Akaike information criterion (AIC), Bayesian information criterion (BIC), Sample-Size Adjusted Bayesian Information Criterion (SS-BIC), Entropy, Lo–Mendell–Rubin Test (LMR), and Bootstrap Likelihood Ratio Test (BLRT). In LCA, the lower values of the AIC, BIC and SS-BIC are preferred. The entropy values range from 0 to 1, with higher values preferred, and cannot be lower than 0.8. The LMRT and BLRT should reach significant level (*p* < 0.05) (Dziak et al., 2014).

### 2.6 Ethical considerations

This study was approved by the Institutional Review Board at the researchers’ university (Grant Number: E202165). Permission to collect data was granted by the principal and head teacher at each school before conducting the survey. The participants were informed of the purpose, method, and considerations of the study and that they could quit at any time during the filling process in the survey. They and their parents or legal guardian(s) further signed an informed consent form. The cover page of the questionnaire contained contact information for psychological consultations, should they need to.

## 3 Results

A total of 2,000 electronic questionnaires were issued, and 1846 questionnaires were returned, for a response rate of 92.3%. The mean age of the 1,846 participants was 10.10 [standard deviation (SD) = 1.82] years (ranging from 6 to 14). Half of the participants were boys (50.8%). A quarter of the participants were in grade 6 (25.3%). Approximately 60.2% of the participants lived in urban areas, and less than a fifth of the participants were only children (16.3%). Nearly a quarter of the participants were left-behind children (22.5%). Most children think their parents have a good relationship (60.5%). More than half of the children said their parents argued little (52.5%). More than half of the participants had a core family structure (54.1%). Most children’s caregivers are mothers (63.6%). Approximately 73.5% of participants had more than three friends of the same sex, while 41.7% had 0–1 friends of the opposite sex.

### 3.1 Latent class analysis

Five models were estimated during exploration, whose fit metrics are shown in Table 1. The LMR value (*p* = 0.197) of the five-class model was non-significant. The probability (7.9%) of one class of the four-class model is less than 10%. The Log(L), AIC, BIC, and SS-BIC values in the three-class model were lower than those of the two-class model, and the entropy value was higher than 0.8, which indicates that the three-class model is better than the two-class model. Overall, the three-class model was optimal, and the fit metrics are highlighted in bold in Table 1.

**Table 1:** Goodness-of-fit statistics for one- to five-class models.

Model	k	Log(L)	AIC	BIC	SS-BIC	Entropy	LMR	BLRT	Probability of class
1 class	30	−35897.643	71855.286	72020.910	71925.601	–	–	–	–
2 classes	61	−30773.341	61668.682	62005.450	61811.654	0.928	0.000	0.000	0.36674/ 0.63326
**3 classes**	**92**	**−29583.553**	**59351.106**	**59859.018**	**59566.737**	**0.888**	**0.000**	**0.000**	**0.20585/0.42308/ 0.37107**
4 classes	123	−29238.424	58722.848	59401.904	59011.136	0.881	0.000	0.000	0.07909/0.33532/0.18310/0.40249
5 classes	154	−29047.423	58402.846	59253.046	58763.792	0.857	0.197	0.000	0.16956/0.13109/0.24973/0.37920/0.07042

k, Number of free parameters; Log(L), Log-likelihood value; AIC, Akaike Information Criterion; BIC, Bayesian Information Criterion; SS-BIC, Sample-Size Adjusted Bayesian Information Criterion; LMR, Lo–Mendell–Rubin Test; BLRT, Bootstrap Likelihood Ratio Test. 3-class model was optimal is the optimal classification.

**Figure 1:**
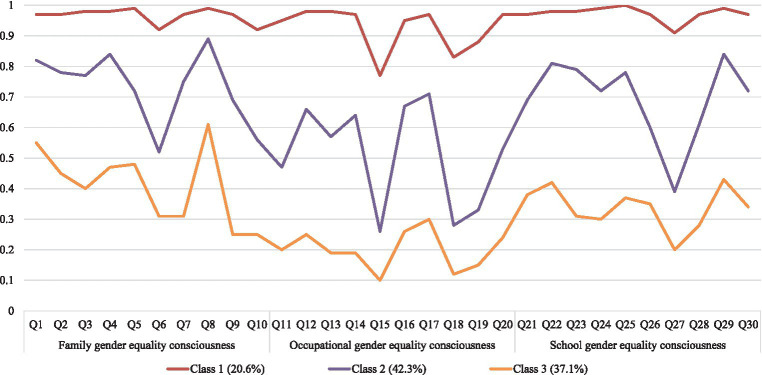
Latent classes of gender equality consciousness among school-age children.

The scores of three classes on 30 items of three dimensions are shown in Figure 1. Class 1 was named the ‘high gender equality consciousness’ group, accounting for 20.6% (*n* = 380) of all participants. It was notable that children in this class reported the highest score for all items. Children in class 2 showed a moderate level of all items, which accounted for 42.3% (*n* = 781) of the sample. Therefore, this subgroup was named “moderate gender equality consciousness.” Class 3 was named the “low gender equality consciousness” group because it had the lowest scores on the most items. Class 3 accounted for 37.1% (*n* = 685) of the sample. The level of gender equality consciousness of different classes of school-age children in various fields is shown in Table 2.

**Table 2:** Gender equality consciousness of school-age children.

Variables	Overall (*N* = 1846)	Class 1 (*N* = 380)	Class 2 (*N* = 781)	Class 3 (*N* = 685)
Overall gender equality consciousness level	0.588	0.955	0.647	0.316
Family gender equality consciousness level	0.661	0.968	0.734	0.408
Occupational gender equality consciousness level	0.481	0.925	0.512	0.201
School gender equality consciousness level	0.620	0.973	0.696	0.338

### 3.2 Demographic characteristics of each class

**Figure 2:**
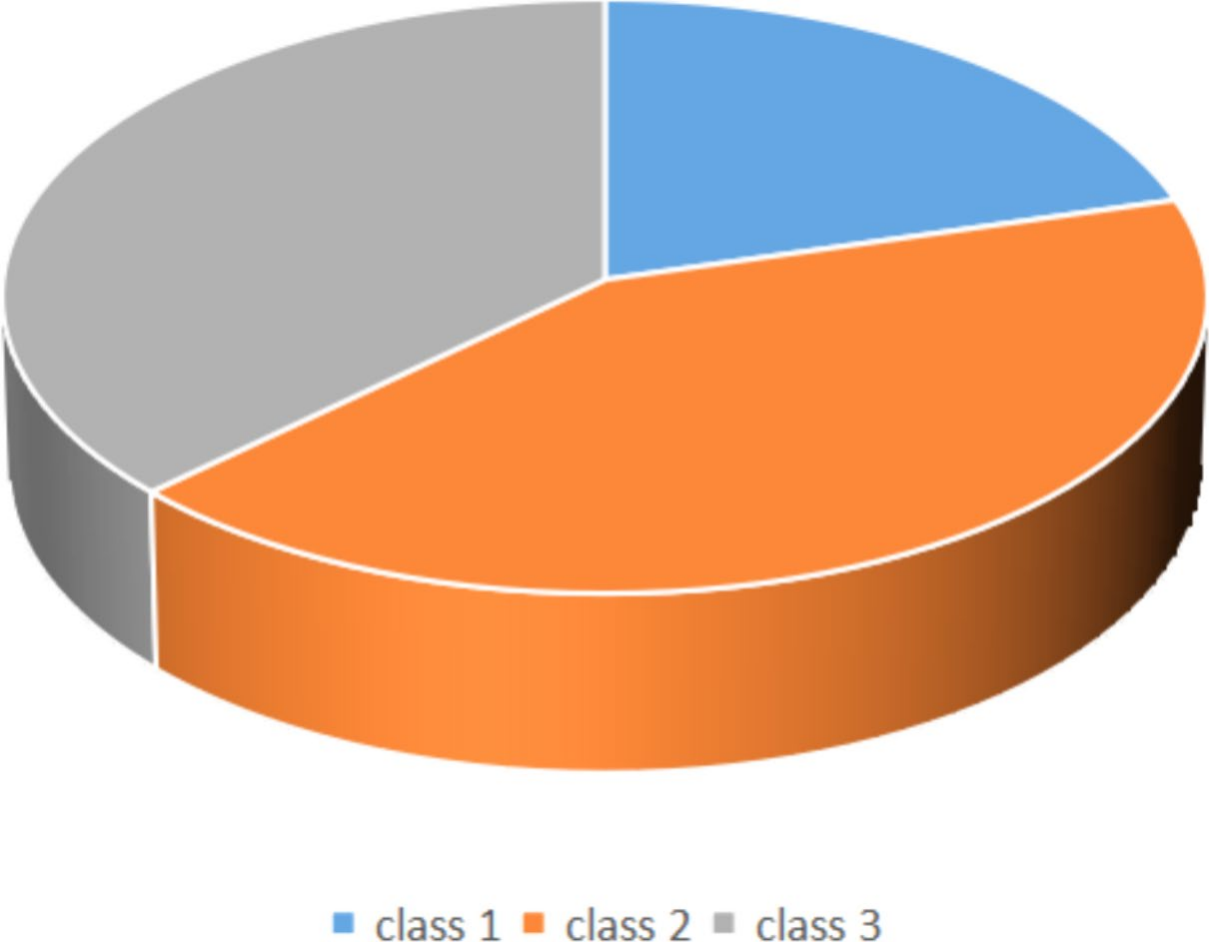
Pie plot of 3 latent classes of gender equality consciousness.

**Figure 3:**
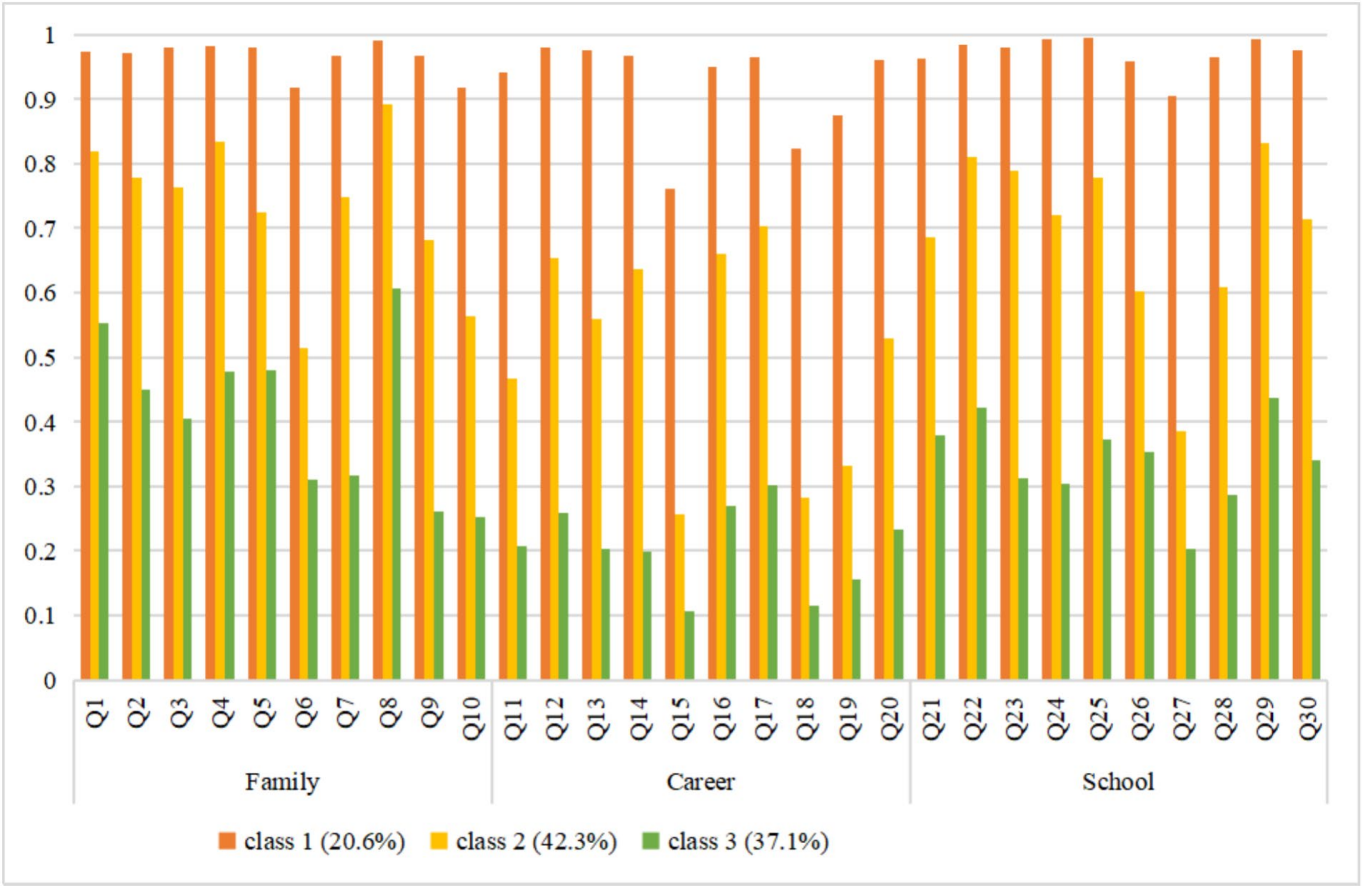
Density plot of 3 latent classes of gender equality consciousness.

The sociodemographic information of participants is presented in Table 3. The “high gender equality consciousness” group accounted for the largest proportion of school-age children who were girls (57.1% vs.50.4% vs. 43.4%), grade 6 (35.8% vs. 30.2% vs. 13.9%), living in urban areas (61.6% vs. 60.1% vs. 59.7%), non-only children (85.8% vs. 82.8% vs. 83.5%), non-left-behind children (86.3% vs. 76.4% vs. 73.9%), parents had a good relationship (70.3% vs. 66.6% vs. 60.3%), core family structure (56.3% vs. 54.5% vs. 52.3%), and the number of same -sex friends was greater than 3 (76.3% vs. 75.5% vs. 69.6%). The “moderate gender equality consciousness” group accounted for the largest proportion of school-age children whose parents argued little (54.5% vs. 47.1% vs. 53.3%), whose caregivers were mothers (65.2% vs. 63.9% vs. 61.6%) and the number of opposite-sex friends was 0–1 (43.1% vs. 40.0% vs. 40.9%) (Figures 2, 3).

**Table 3:** Sociodemographic information of participants (*n* = 1,846).

Variables	Overall (*N* = 1846) N (%)	Class 1 (*N* = 380) N (%)	Class 2 (*N* = 781) N (%)	Class 3 (*N* = 685) N (%)
Gender				
Boy	938(50.8)	163(42.9)	387(49.6)	388(56.6)
Girl	908(49.2)	217(57.1)	394(50.4)	297(43.4)
Grade				
Grade 1	229(12.4)	23(6.1)	61(7.8)	145(21.2)
Grade 2	270(14.6)	36(9.5)	107(13.7)	127(18.5)
Grade 3	246(13.3)	38(10.0)	95(12.2)	113(16.5)
Grade 4	298(16.1)	35(9.2)	129(16.5)	134(19.6)
Grade 5	336(18.2)	112(29.5)	153(19.6)	71(10.4)
Grade 6	467(25.3)	136(35.8)	236(30.2)	95(13.9)
Residence				
Urban	1,112(60.2)	234(61.6)	469(60.1)	409(59.7)
Rural	734(39.8)	146(38.4)	312(39.9)	276(40.3)
The only child				
Yes	301(16.3)	54(14.2)	134(17.2)	113(16.5)
No	1,545(83.7)	326(85.8)	647(82.8)	572(83.5)
Left-behind children				
Yes	415(22.5)	52(13.7)	184(23.6)	179(26.1)
No	1,431(77.5)	328(86.3)	597(76.4)	506(73.9)
Parents’ relationship				
Good	1,200(65.0)	267(70.3)	520(66.6)	413(60.3)
Average	481(26.1)	95(25.0)	189(24.2)	197(28.8)
Bad	76(4.1)	9(2.4)	30(3.8)	37(5.4)
Divorce	89(4.8)	9(2.4)	42(5.4)	38(5.5)
Parental quarrel				
Never	764(41.4)	179(47.1)	319(40.8)	266(38.8)
Little	970(52.5)	179(47.1)	426(54.5)	365(53.3)
Many	112(6.1)	22(5.8)	36(4.6)	54(7.9)
Family structure				
Core family	998(54.1)	214(56.3)	426(54.5)	358(52.3)
Linear family	511(27.7)	104(27.4)	215(27.5)	192(28.0)
Left-behind family	144(7.8)	21(5.5)	55(7.0)	68(9.9)
Single parent family	66(3.6)	10(2.6)	27(3.5)	29(4.2)
Other	127(6.9)	31(8.2)	58(7.4)	38(5.5)
Caregiver				
Father	157(8.5)	37(9.7)	51(6.5)	69(10.1)
Mother	1,174(63.6)	243(63.9)	509(65.2)	422(61.6)
Grandparents	412(22.3)	69(18.2)	168(21.5)	175(25.5)
Siblings	103(5.6)	31(8.2)	53(6.8)	19(2.8)
Number of same -sex friends				
0–1	177(9.6)	28(7.4)	64(8.2)	85(12.4)
2–3	312(16.9)	62(16.3)	127(16.3)	123(18.0)
>3	1,357(73.5)	290(76.3)	590(75.5)	477(69.6)
Number of opposite-sex friends				
0–1	769(41.7)	152(40.0)	337(43.1)	280(40.9)
2–3	453(24.5)	93(24.5)	186(23.8)	174(25.4)
>3	624(33.8)	135(35.5)	258(33.0)	231(33.7)

Core family: A family unit consisting of parents and children. Linear family: A family unit consisting of grandparents, parents and children. Left-behind family: Parents go out to work, grandparents and children live in the residence. Single parent family: A family unit consisting of one parent and children.

### 3.3 Predictor of latent class membership

To identify the predictors of class membership, a multinomial logistic regression was conducted with the “low gender equality consciousness” group as the reference. The Predictors are highlighted in bold in Table 4. The school-age children who were girls, grade 6 and whose caregivers were siblings were more likely to be in the “high gender equality consciousness” and “moderate gender equality consciousness” group compared with those in the “low gender equality consciousness” group. Rural children are more likely to be in the “low gender equality consciousness” group than urban children. Left-behind children were more likely to be in the “low gender equality consciousness” group compared with those in the “high gender equality consciousness” group. Children whose parents have a good relationship were more likely to be in the “low gender equality consciousness” group compared with those in the “high gender equality consciousness” group.

**Table 4:** Predictor of latent class membership.

	B	SE	OR	95% confidence interval	*p*
Class 1: High gender equality consciousness (vs. Class 3: Low gender equality consciousness)					
Constant	1.019	0.676	-	-	0.132
**Gender: boy, ref.: girl**	**−0.651**	**0.141**	**0.522**	**0.395–0.688**	**0.000**
**Grade: Grade 1, ref.: Grade 6**	**−2.479**	**0.280**	**0.084**	**0.048–0.145**	**0.000**
**Grade: Grade 2, ref.: Grade 6**	**−1.903**	**0.246**	**0.149**	**0.092–0.242**	**0.000**
**Grade: Grade 3, ref.: Grade 6**	**−1.661**	**0.245**	**0.190**	**0.117–0.307**	**0.000**
**Grade: Grade 4, ref.: Grade 6**	**−1.928**	**0.244**	**0.145**	**0.090–0.235**	**0.000**
Grade: Grade 5, ref.: Grade 6	0.006	0.212	1.006	0.664–1.523	0.978
**Residence: Urban, ref.: Rural**	**0.455**	**0.162**	**1.576**	**1.148–2.163**	**0.005**
The only child: Yes, ref.: No	0.090	0.200	1.095	0.739–1.622	0.652
**Left-behind children: Yes, ref.: No**	**−0.587**	**0.193**	**0.556**	**0.381–0.811**	**0.002**
**Parents’ relationship: Good, ref.: Divorce**	**1.237**	**0.494**	**3.446**	**1.307–9.081**	**0.012**
Parents’ relationship: Average, ref.: Divorce	0.931	0.500	2.537	0.952–6.761	0.063
Parents’ relationship: Bad, ref.: Divorce	0.364	0.627	1.440	0.421–4.923	0.561
Parental quarrel: Never, ref.: Many	0.053	0.327	1.055	0.555–2.004	0.871
Parental quarrel: Few, ref.: Many	−0.246	0.317	0.782	0.420–1.456	0.438
Family structure: Core family, ref.: Other	−0.320	0.298	0.726	0.405–1.301	0.282
Family structure: Linear family, ref.: Other	−0.259	0.305	0.772	0.425–1.404	0.396
Family structure: Left-behind family, ref.: Other	−0.485	0.403	0.616	0.280–1.357	0.229
Family structure: Single parent family, ref.: Other	−0.140	0.546	0.869	0.298–2.534	0.798
**Caregiver: Father, ref.: Siblings**	**−0.895**	**0.396**	**0.409**	**0.188–0.887**	**0.024**
**Caregiver: Mother, ref.: Siblings**	**−1.050**	**0.338**	**0.350**	**0.180–0.679**	**0.002**
**Caregiver: Grandparents, ref.: Siblings**	**−1.067**	**0.364**	**0.344**	**0.169–0.702**	**0.003**
Number of same -sex friends: 0–1, ref.:>3	−0.107	0.252	0.898	0.548–1.472	0.670
Number of same -sex friends: 2–3, ref.:>3	0.049	0.192	1.051	0.721–1.531	0.798
Number of opposite-sex friends: 0–1, ref.:>3	−0.084	0.166	0.919	0.664–1.272	0.612
Number of opposite-sex friends: 2–3, ref.:>3	−0.070	0.186	0.932	0.647–1.343	0.707
Class 2: Moderate gender equality consciousness (vs. Class 3: Low gender equality consciousness)					
Constant	1.561	0.502	–	–	0.002
**Gender: boy, ref.: girl**	**−0.353**	**0.112**	**0.703**	**0.564–0.875**	**0.002**
**Grade: Grade 1, ref.: Grade 6**	**−1.938**	**0.211**	**0.144**	**0.095–0.218**	**0.000**
**Grade: Grade 2, ref.: Grade 6**	**−1.265**	**0.190**	**0.282**	**0.195–0.410**	**0.000**
**Grade: Grade 3, ref.: Grade 6**	**−1.223**	**0.195**	**0.294**	**0.201–0.432**	**0.000**
**Grade: Grade 4, ref.: Grade 6**	**−1.086**	**0.182**	**0.337**	**0.236–0.482**	**0.000**
Grade: Grade 5, ref.: Grade 6	−0.191	0.193	0.826	0.566–1.206	0.322
**Residence: Urban, ref.: Rural**	**0.320**	**0.130**	**1.377**	**1.066–1.778**	**0.014**
The only child: Yes, ref.: No	0.245	0.155	1.277	0.943–1.729	0.113
Left-behind children: Yes, ref.: No	0.018	0.137	1.018	0.778–1.332	0.898
Parents’ relationship: Good, ref.: Divorce	0.090	0.314	1.095	0.591–2.027	0.773
Parents’ relationship: Average, ref.: Divorce	−0.185	0.321	0.831	0.443–1.559	0.564
Parents’ relationship: Bad, ref.: Divorce	−0.095	0.398	0.909	0.417–1.982	0.810
Parental quarrel: Never, ref.: Many	0.412	0.264	1.509	0.899–2.534	0.120
Parental quarrel: Few, ref.: Many	0.426	0.254	1.531	0.930–2.521	0.094
Family structure: Core family, ref.: Other	−0.149	0.248	0.861	0.530–1.400	0.547
Family structure: Linear family, ref.: Other	−0.120	0.253	0.887	0.541–1.456	0.636
Family structure: Left-behind family, ref.: Other	−0.534	0.316	0.586	0.315–1.089	0.091
Family structure: Single parent family, ref.: Other	−0.352	0.405	0.703	0.318–1.555	0.385
**Caregiver: Father, ref.: Siblings**	**−1.230**	**0.347**	**0.292**	**0.148–0.577**	**0.000**
**Caregiver: Mother, ref.: Siblings**	**−0.820**	**0.297**	**0.440**	**0.246–0.789**	**0.006**
**Caregiver: Grandparents, ref.: Siblings**	**−0.843**	**0.312**	**0.430**	**0.233–0.794**	**0.007**
Number of same -sex friends: 0–1, ref.:>3	−0.184	0.190	0.832	0.574–1.206	0.331
Number of same -sex friends: 2–3, ref.:>3	−0.031	0.152	0.970	0.719–1.307	0.840
Number of opposite-sex friends: 0–1, ref.:>3	0.060	0.133	1.062	0.819–1.378	0.649
Number of opposite-sex friends: 2–3, ref.:>3	−0.006	0.150	0.994	0.741–1.333	0.966

Bold text indicates that the variable is significant.

### 3.4 Gender role with latent class membership

Chi-square test was used to explore the differences in gender roles among the three classes of school-age children (Table 5). The frequency of androgynous school-age children in classes 1, 2, and 3 were 293 (77.1%), 570 (73.0%), 450 (65.7%), respectively. As shown in Table 5, the gender roles of school-age children statistically differed across the three classes (*p* < 0.05). Moreover, the pairwise comparison revealed that the proportion of school-age children with femininity in the “low gender equality consciousness” group was significantly higher than that in the “moderate gender equality consciousness” group (*p* < 0.05). And the proportion of androgynous school-age children in the “low gender equality consciousness” group was significantly lower than that in the “moderate gender equality consciousness” and “moderate gender equality consciousness” group (p < 0.05).

**Table 5:** Gender role difference of three classes.

		Class 1 (*n* = 380)	Class 2 (*n* = 781)	Class 3 (*n* = 685)	χ^2^	*P*
Undifferentiated (*n* = 178)	n	29_a_	74_a_	75_a_	22.969	0.000
	%	7.6	9.5	10.9		
Masculinity (*n* = 106)	n	13_a_	50_a_	43_a_		
	%	3.4	6.4	6.4		
Femininity (*n* = 249)	n	45_a, b_	87_b_	117_a_		
	%	11.8	11.1	17.1		
Androgynous (*n* = 1,313)	n	293_a_	570_a_	450_b_		
	%	77.1	73.0	65.7		

The same subscript letter indicates that there is no significant difference between the column proportions of these categories at the 0.05 level.

## 4 Discussion

### 4.1 Latent classes of gender equality consciousness

By taking a person-centered approach to analyze the gender equality consciousness of school-age children, this study aimed to highlight the differences in their gender equality consciousness and to guide further research on tailored gender equality consciousness improvement according to latent classes. To the best of the authors’ knowledge, this study is the first to use LCA to identify the latent classes of gender equality consciousness among school-age children, hence complementing previous studies that treat school-age children as a homogeneous whole. Therefore, this study helps to develop targeted intervention measures according to the characteristics of the different gender equality consciousness classes of children.

The findings of this study revealed the distinct categorical features of the gender equality consciousness among school-age children. Based on the score responses for each item, three classes were identified, namely, the “high gender equality consciousness,” “moderate gender equality consciousness” and “low gender equality consciousness” groups. This classification reflects the heterogeneity of school-age children in each latent class and can be used as a reference for comparison in the future.

The “high gender equality consciousness” group consisted of 20.6% of the sample. School-age children in this subgroup had the highest scores of all items among the three subgroups. This result indicates that children in this subgroup have the best gender equality consciousness in family, occupation and school. Nevertheless, the response of children in this subgroup to some of the items was notable. For example, in the family field, the subgroup’s level of gender equality consciousness in item 6 “Who do you think should be mainly responsible for working and earning money?” and item 10 “Who do you think should be mainly responsible for managing family property?” was lower than the average level of family gender equality consciousness among the subgroup’s children. In the occupational field, the level of gender equality consciousness in item 15 “Who do you think is more suitable for kindergarten teacher?” item 18 “Who do you think is more suitable for driver?” and item 19 “Who do you think is more suitable for work requiring attention to details?” was lower than the average level of occupational gender equality consciousness of children in this subgroup. In the school field, the level of gender equality consciousness in item 27 “Who do you think should be more active in class sports?” was lower than the average level of school gender equality consciousness of children in this subgroup. This was also the case for children in the “moderate gender equality consciousness” group and the “moderate gender equality consciousness” group. This suggests that the relative unequal cognition of the above six items belongs to the common problem of school-age children, which may be caused by the common collective phenomena and collective cognition in the social environment where school-age children live (Yang and Zhang, 2019). However, the perception of gender inequality in each item means a limitation on children’s future life, study and work choices, preventing them from fully exploring and developing their potential. Therefore, gender equality education policy makers and implementers pay particular attention to these aspects.

Firstly, gender equality educators should help children establish equal gender cognition in family finance and not let them be influenced by social environment. Secondly, in the occupational field, educators should tell children that any occupation, including drivers and kindergarten teachers, should not be limited by gender, so as to break the cognitive limits of children’s career development. Finally, in the school field, educators can make children realize that sportsmanship is unisex, and everyone can actively cope with sports activities through extra-curricular activities.

The “moderate gender equality consciousness” group has the highest proportion of 42.3%. The average level of the gender equality consciousness of school-age children in this subgroup is 0.647, which is the group closest to the overall level of school-age children. In addition to the common problems demonstrated by the “high gender equality consciousness” group, the following aspects also require special attention in this subgroup of children. In the family field, the level of gender equality consciousness of children in item 5 “Who do you think should be the main care of children’s life?” and item 9 “Who do you think is the final decision when buying a house?” was lower than the average level of family gender equality consciousness of children in this subgroup. In the occupational field, the level of gender equality consciousness in item 11 “Who do you think is more suitable for secretary work?” was lower than the average level of occupational gender equality consciousness of children in this subgroup. In the school field, the level of gender equality consciousness in item 26 “Who do you think should get more care from teachers in outdoor activities?” and “item 2” Who do you think should do more work in health work?’ was lower than the average level of school gender equality consciousness of children in this subgroup. This suggests that the formulation and implementation of gender equality education policies, when dealing with this subgroup of children, should take care to teach them that men and women are equal in the family, and that they should have the same rights and obligations, whether in childcare or in any decision-making. It is also important to teach such children that it is their personality and preferences, not their sex, that determine their suitability for a particular job. In the school field, educators can help children to reflect on and correct erroneous gender stereotypes by giving them a practical experience of gender equality through the organization of outdoor activities and classroom cleaning.

The “low gender equality consciousness” group consisted of 37.1% of the sample, indicates that there are currently a large number of children in China with a low gender equality consciousness, and that gender equality education needs to be urgently put on the agenda. The following items are exclusive to this subgroup of children. In the family field, the level of gender equality consciousness of children in item 3 “Who do you think is more appropriate to pick up and drop off the children?” was lower than the average level of family gender equality consciousness of children in this subgroup. In the occupational field, the level of children’s gender equality consciousness of this subgroup in the items 13 “Who do you think is more suitable for a competitive occupation?” and 14 “Who do you think is more suitable for a job that requires a high level of thinking?” is lower than the average level of children’s occupational gender equality consciousness in this subgroup. In the school field, the level of gender equality consciousness in item 23 “Who do you think should be questioned more often when there are difficult questions in the English lessons?” and item 24 “Who do you think should be questioned more often when there are difficult questions in the math lessons?” was lower than the average level of school gender equality consciousness of children in this subgroup.

Although the gender equality awareness of school-age children is divided into three classes through the LCA, there are common problems in the family, occupation and school fields, that is, women are more suitable for taking care of the family, non-creative work and non-physical school activities. The formation of gender equality consciousness is affected by various factors. With the development of electronic media, a large number of television advertising spread to school-age children. In television advertising across the globe, the odds of women taking on household duties are approximately 3.5 times higher than for men (Eisend, 2010)^.^ The products endorsed by women are mainly cosmetics and personal care, while those endorsed by men are mostly automobiles and digital products (Matthes et al., 2016). Students are likely to internalize the division labor of occupation and household into their own consciousness. They understand the phenomenon seen as “should” or “more appropriate,” instead of analyzing the abilities and differences between men and women from an objective perspective. Therefore, when the policy makers and implementer of gender equality education face the children in the “low gender equality consciousness” group, they can first carry out family activities or occupational role playing in school, so that children can realize the equality of men and women in family and workplace when they get along with other people. Secondly, it is possible to carry out thinking activities or watch an educational video about gender equality in schools in which the concept of gender equality is conveyed to school-age children. Lastly, integration classes can be used in math, English and other subjects, where teachers can incorporate the concept of gender equality into the education of children by asking questions, praising and encouraging them on an equal footing, in order to reverse the false gender stereotypes of the children.

### 4.2 Demographic characteristics of each class

Demographic predictors of class membership include gender, grade, caregiver, place of residence, whether they are left-behind children and parental relationship. Children who are girls, in higher grades, and whose caregivers are siblings, and their parents have a good relationship are less likely to enter the “low gender equality consciousness” group. Rural and left-behind children are more likely to enter the “low gender equality consciousness” group. This may be due to the fact that females, as victims of gender inequality, usually desire or pursue gender equality more than males. Coupled with the fact that school-age children are still at a developmental level of thinking. Studies have found that school-age girls usually develop their gender identity earlier than boys, and have a stronger sense of identification with the opposite sex’s gender traits (Fen, 2007). Therefore, it is possible that girls at this age also have a higher sense of gender equality due to a higher level of maturity in gender identity development, which makes it easier for them to identify and recognize the good qualities of the opposite sex and the limitations of stereotypical knowledge of gender. As a result of school education and their own cognitive growth, children in the upper grades are gradually becoming more mature in their understanding of gender, and no longer need to use stereotypical knowledge to distinguish between boys and girls (Ruble et al., 2007). In addition, school education and peer interactions may break down their original stereotypes, and thus a more equal gender consciousness will easily emerge (Xin, 2006). Children’s siblings, as growers in the new era, may be influenced by the social environment in which China’s basic national policy of gender equality is constantly being improved, and thus have a more equal gender consciousness than their parents and grandparents who are deeply influenced by old patriarchal ideas, which will have a more positive guiding effect on children’s gender equality consciousness (Zhang, 2019). The results of Cao’s study also confirmed that equal gender education attitudes are more likely to reduce children’s gender stereotypes (Cao, 2010). A good relationship between parents may mean that the relationship between parents is more equal and there is less gender inequality. Nowadays, besides mother and father, the concept of family is becoming more inclusive, covering same gender couples, multi-generation homes, single family households, and others. For example, lesbian couples is more equal than heterosexual couples in distribution of household labor, which may affect children’s view of gender equality. Moreover, with insufficient understanding of gender equality, the education for children by older adult can lead to the aggravation of their gender role stereotypes. Thus, a family environment with sufficient gender equality consciousness may contribute to the generation of gender equality consciousness of school-age children (Liu, 2022).

The low gender equality consciousness among rural and left-behind children may be due to the fact that they live in a gender-unequal family and social environment. Previous study has found that the concept of gender equality is generally low in rural areas of China, especially among the older adult (Hou, 2022). The older adult are the primary caregivers of left-behind children. Therefore, in the formulation and implementation of gender equality education programs, these children should be given priority attention. Educators should not only formulate targeted educational programs based on their psychological characteristics, but also pay close attention to the psychological changes of these children when implementing them, so as to make timely adjustments and interventions.

### 4.3 Gender role of the three classes

The results show that the proportion of children with feminine gender role was significantly higher in the “low gender equality consciousness” group than in the “moderate gender equality consciousness” group. The proportion of children with androgynous gender role in the “low gender equality consciousness” group was significantly lower than that in the “moderate gender equality consciousness” and “high gender equality consciousness” group. Children with low gender equality consciousness may tend to suffer higher gender role stress and have a lower self-esteem, such as left-behind children. Masculine gender role is attenuated, which is characterized by failing to be tough, letting someone else take control, and lack of independence (Harrington et al., 2022).^.^On the one hand, this suggests that gender role is a significant predictor of gender equality consciousness among school-age children. Gender equality educators are reminded to focus on feminized children. For example, they can cultivate their masculine characteristics through methods such as adding setbacks in the process of education and role-playing, so as to achieve the educational goal of helping children to shape androgynous gender role and equal gender concepts for their healthy growth. On the other hand, gender role may be an important intervention target for school-age children’s gender equality consciousness. It suggests that the formulators and implementers of gender equality education policies should pay attention to the importance of bisexual education. For example, they can make use of group games and physical exercises to increase children’s contact with the opposite sex and help them to understand and learn from each other’s strengths. This will help children deepen their own gender strengths while learning from each other’s good qualities, and help them develop androgynous gender role that combine the good qualities of both men and women.

## 5 Limitations

There are several limitations of this study that should be acknowledged. Firstly, this study was only carried out in Henan Province of China, which has some bias, and the sample source area should be expanded in the future. Next, the study is a cross-sectional study and identification of causal relationships between variables may not be possible. Therefore, further longitudinal studies are recommended to follow up the trajectory of gender equality consciousness among children.

## 6 Conclusion

Chinese children’s gender equality consciousness is generally at a moderate or low level, with fewer children having a high gender equality consciousness. When developing targeted interventions for school-age children’s gender equality consciousness, educators should pay attention to the characteristics of each class. As the LCA results show, rural children and left-behind children are the priority groups for gender equality education. Gender role is the important predictors and intervention targets of children’s gender equality consciousness. When designing and implementing gender equality education programs, educators should focus on children with feminine gender role, and at the same time incorporate the concept of bisexual education into the education process, so as to help school-age children develop androgynous gender role with good qualities of both boys and girls, and thus improve their gender equality consciousness.

## Data availability statement

The raw data supporting the conclusions of this article will be made available by the authors, without undue reservation.

## Ethics statement

The studies involving humans were approved by the Xiangya Nursing School of Central South University. The studies were conducted in accordance with the local legislation and institutional requirements. Written informed consent for participation in this study was provided by the participants’ legal guardians/next of kin.

## Author contributions

YL: Conceptualization, Data curation, Investigation, Writing – original draft, Writing – review & editing. JPZ: Conceptualization, Funding acquisition, Writing – original draft, Writing – review & editing. JZ: Conceptualization, Supervision, Writing – original draft. JL: Data curation, Investigation, Writing – review & editing. YC: Data curation, Investigation, Writing – review & editing. MZ: Funding acquisition, Supervision, Writing – original draft, Writing – review & editing.

## References

[ref1] Banse R. Gawronski B. Rebetez C. Gutt H. Morton J. B. (2010). The development of spontaneous gender stereotyping in childhood: relations to stereotype knowledge and stereotype flexibility. Dev. Sci. 13, 298–306. doi: 10.1111/j.1467-7687.2009.00880.x, PMID: 20136926

[ref2] Bem S. L. (1974). The measurement of psychological androgyny. J. Consult. Clin. Psychol. 42, 155–162. doi: 10.1037/h0036215 4823550

[ref3] Bem S. L. Estes W. K. (1981). Gender schema theory: a cognitive account of sex typing. Psychol. Rev. 88, 354–364. doi: 10.1037/0033-295X.88.4.354

[ref4] Bleidorn W. Arslan R. C. Denissen J. J. Rentfrow P. J. Gebauer J. E. Potter J.. (2016). Age and gender differences in self-esteem-a cross-cultural window. J. Pers. Soc. Psychol. 111, 396–410. doi: 10.1037/pspp0000078, PMID: 26692356

[ref5] Bourdieu P. (2004). Practice and reflection: a guide to reflective sociology. Beijing: Central Compilation Press.

[ref6] Cao R. Y. (2010). The relationship between the development of children's gender stereotypes and gender constancy: the adjustment of mothers' parenting attitudes. Jinan: Shandong Normal University.

[ref7] Cui S. Cheng F. Zhang L. Zhang C. Yuan Q. Huang C.. (2021). Self-esteem, social support and coping strategies of left-behind children in rural China, and the intermediary role of subjective support:a cross-sectional survey. BMC Psychiatry 21:158. doi: 10.1186/s12888-021-03160-y, PMID: 33731074 PMC7972224

[ref8] Dalmartello M. Decarli A. Ferraroni M. Bravi F. Serraino D. Garavello W.. (2020). Dietary patterns and oral and pharyngeal cancer using latent class analysisi. Int. J. Cancer 147, 719–727. doi: 10.1002/ijc.32769, PMID: 31677269

[ref9] Ding C. S. (2018). Fundamentals of applied multidimensional scaling for educational and psychological research. Cham: Springer International Publishing AG.

[ref10] Duan C. Zhou F. (2005). Research on the left-behind children in China. Popul. Res. 1, 29–36.

[ref11] Dziak J. Lanza S. T. Tan X. (2014). Effect size, statistical power and sample size requirements for the bootstrap likelihood ratio test in latent class analysis. Struct. Equ. Modeling 21, 534–552. doi: 10.1080/10705511.2014.919819, PMID: 25328371 PMC4196274

[ref12] Eisend M. (2010). A meta-analysis of gender roles in advertising. J. Acad. Mark. Sci. 38, 418–440. doi: 10.1007/s11747-009-0181-x

[ref13] Fen M. Z. Liao Z. F. (2007). Investigation on gender identity of primary school children. J. Changzhou Inst. Technol. 2, 36–42. doi: 10.3969/j.issn.1673-0887.2007.02.009

[ref14] Geng X. W. Zhang F. (2012). The relationship between gender roles and subjective well-being of college students: the mediating role of self-esteem. Psychol. Behav. Res. 10, 384–388.

[ref15] Harrington A. G. Overall N. C. Maxwell J. A. (2022). Feminine gender role discrepancy strain and Women's self-esteem in daily and weekly life: a person x context perspective. Sex Roles 87, 35–51. doi: 10.1007/s11199-022-01305-1, PMID: 35729999 PMC9189801

[ref16] Heinz A. Catunda C. van Duin C. Torsheim T. Willems H. (2020). Patterns of health-related gender inequalities-a cluster analysis of 45 countries. J. Adolesc. Health 66, S29–S39. doi: 10.1016/j.jadohealth.2020.02.011, PMID: 32446606

[ref17] Hou X. Y. (2022). Research on multidimensional relative poverty and its influencing factors from the perspective of gender difference. Wuhan: Zhongnan University of Economics and Law.

[ref18] Kolip P. Lange C. Finne E. (2019). Gender equality and the gender gap in life expectancy in Germany. Bundesgesundheitsblatt Gesundheitsforschung Gesundheitsschutz 62, 943–951. doi: 10.1007/s00103-019-02974-2 31165173

[ref19] Li Y. Zuo M. Peng Y. Zhang J. Chen Y. Tao Y.. (2021). Gender differences influence gender equality awareness, self-esteem, and subjective well-being among school-age children in China. Front. Psychol. 12:671785. doi: 10.3389/fpsyg.2021.671785 35095630 PMC8795625

[ref20] Liu A. Y. (2022). Domestic labor division between husband and wife under the interaction of relative resources and gender role concepts. J. Chin. Wome. Coll. 2, 27–35. doi: 10.13277/j.cnki.jcwu.2022.02.005

[ref21] Liuji G. (2009). Bourdieu's theory of social practice. Kaifeng: Henan University Press.

[ref22] Lu Q. Su Y. J. (2003). Investigation and revision of the Bem sex role scale. Chin. J. Ment. Health 8, 550–553. doi: 10.3321/j.issn:1000-6729.2003.08.012

[ref23] Luijten C. C. van de Bongardt D. Nieboer A. P. (2023). Adolescents' friendship quality and over-time development of well-being: the explanatory role of self-esteem. J. Adolesc. 95, 1057–1069. doi: 10.1002/jad.12175, PMID: 37042634

[ref24] Ma C. Liu Y. F. Wu X. G. (2023). The categorical characteristics of adolescent future orientation and its relationship with career development. Chin. J. Ment. Health 10, 866–872. doi: 10.3969/j.issn.1000-6729.2023.10.007

[ref25] Matthes J. Prieler M. Adam K. (2016). Gender-role portrayals in television advertising across the globe. Sex Roles 75, 314–327. doi: 10.1007/s11199-016-0617-y, PMID: 27688526 PMC5023740

[ref26] Matud M. P. López-Curbelo M. Fortes D. (2019). Gender and psychological well-being. Int. J. Environ. Res. Public Health 16:3531. doi: 10.3390/ijerph16193531, PMID: 31547223 PMC6801582

[ref27] Meyer J. P. Morin A. J. S. (2016). A person-centered approach to commitment research: theory, research, and methodology. J. Organ. Behav. 37, 584–612. doi: 10.1002/job.2085

[ref28] Milner A. Scovelle A. J. Hewitt B. Maheen H. Ruppanner L. King T. L. (2020). Shifts in gender equality and suicide: a panel study of changes over time in 87 countries. J. Affect. Disord. 276, 495–500. doi: 10.1016/j.jad.2020.07.105, PMID: 32871680

[ref29] Qu S. W. (2022). Analysis on China's gender equality education policy. University 5, 25–28. doi: 10.3969/j.issn.1673-7164.2022.05.007

[ref30] Ruble D. N. Martin C. L. Berenbaum S. A. (2007). “Gender development” in Handbook of child psychology: social, emotional, and personality development. eds. Eisenberg N. Damon W. Lerner R. M.. 6th ed (New York, NY: John Wiley & Sons, Inc), 858–932.

[ref31] Shen Q. J. Lu Y. W. Hu C. Y. Deng X. M. Gao H. Huang X. Q.. (1998). A preliminary study of the mental health of young migrant workers in Shenzhen. Psychiatry Clin. Neurosci. 52, S370–S373. doi: 10.1111/j.1440-1819.1998.tb03272.x, PMID: 9895197

[ref32] Siyanova-Chanturia A. Warren P. Pesciarelli F. Cacciari C. (2015). Gender stereotypes across the ages: on-line processing in school-age children, young and older adults. Front. Psychol. 6:1388. doi: 10.3389/fpsyg.2015.01388 26441763 PMC4585124

[ref33] Su Y. Cui C. Y. Xu D. L. (2020). Current status and influencing factors of gender equality awareness of children and adolescents. Mental Health Educ. Prim. Second. Sch. 25, 4–7. doi: 10.3969/j.issn.1671-2684.2020.25.002

[ref34] Tang W. Gai X. S. Zhao Y. (2011). Survey on the status quo of gender equality awareness among children and adolescents. J. Inner Mong. Norm. Unive. 40, 139–144. doi: 10.3969/j.issn.1001-7623.2011.02.028

[ref35] Valtolina G. G. Colombo C. (2012). Psychological well-being, family relations, and developmental issues of children left behind. Psychol. Rep. 111, 905–928. doi: 10.2466/21.10.17.PR0.111.6.905-928, PMID: 23402056

[ref36] Wang W. P. (2013). Pediatrics. Beijing: People's Medical Publishing House.

[ref37] Wang X. Ling L. Su H. Cheng J. Jin L. Sun Y. H. (2015). Self-concept of left-behind children in China: a systematic review of the literature. Child Care Health Dev. 41, 346–355. doi: 10.1111/cch.12172, PMID: 25039693

[ref38] Xin Z. (2006). The development of Children's gender stereotypes and their impact on social judgment. Shandong: Shandong Normal University.

[ref39] Yang H. Zhang Z. Y. (2019). The changing trend of gender composition in Chinese industry in the past 40 years: equality or segregation? Popul. Econ. 4, 122–134. doi: 10.3969/j.issn.1000-4149.2019.04.009

[ref40] Yu K. Liao Y. Fu D. Chen S. D. Long Q. S. Xu P.. (2020). Androgyny eliminates sex differences in emotional reactivity: ERP and network coupling evidences. Neurosci. Lett. 720:134776. doi: 10.1016/j.neulet.2020.134776, PMID: 31978498

[ref41] Zhang X. B. (2019). Research on the historical evolution and promotion strategy of China's basic national policy of gender equality. Changchun: Northeast Normal University.

[ref42] Zhang J. T. Zhang M. Q. Li G. M. (2017). Follow-up analysis of potential profile model: comparing the deviation after improved classification analysis. New Res. Psychol. 37, 434–440.

